# Flotillins Directly Interact with γ-Catenin and Regulate Epithelial Cell-Cell Adhesion

**DOI:** 10.1371/journal.pone.0084393

**Published:** 2013-12-31

**Authors:** Nina Kurrle, Frauke Völlner, Rüdiger Eming, Michael Hertl, Antje Banning, Ritva Tikkanen

**Affiliations:** 1 Institute of Biochemistry, Medical Faculty, Justus Liebig University, Giessen, Germany; 2 Department of Dermatology and Allergology, Phillips University, Marburg, Germany; University of Birmingham, United Kingdom

## Abstract

Flotillin-1 and flotillin-2 are two homologous, membrane raft associated proteins. Although it has been reported that flotillins are involved in cell adhesion processes and play a role during breast cancer progression, thus making them interesting future therapeutic targets, their precise function has not been well elucidated. The present study investigates the function of these proteins in cell-cell adhesion in non-malignant cells. We have used the non-malignant epithelial MCF10A cells to study the interaction network of flotillins within cell-cell adhesion complexes. RNA interference was used to examine the effect of flotillins on the structure of adherens junctions and on the association of core proteins, such as E-cadherin, with membrane rafts. We here show that the cadherin proteins of the adherens junction associate with flotillin-2 in MCF10A cells and in various human cell lines. *In vitro*, flotillin-1 and flotillin-2 directly interact with γ-catenin which is so far the only protein known to be present both in the adherens junction and the desmosome. Mapping of the interaction domain within the γ-catenin sequence identified the Armadillo domains 6–8, especially ARM domain 7, to be important for the association with flotillins. Furthermore, depletion of flotillins significantly influenced the morphology of the adherens junction in human epithelial MCF10A cells and altered the association of E-cadherin and γ-catenin with membrane rafts. Taken together, these observations suggest a functional role for flotillins, especially flotillin-2, in cell-cell adhesion in non-malignant epithelial cells.

## Introduction

Cell-cell adhesion is based on various cellular junctions and ensures a tight contact between neighboring cells, enabling interactive exchanges necessary for morphological and functional differentiation and maintaining the homeostasis of healthy tissue organization (reviewed in [1]). Particularly during tumor progression, the loss of cell-cell adhesion plays an important role in epithelial-mesenchymal transition (EMT) and metastasis formation (see e.g. [2], [3]). Two important types of cell-cell junctions are the adherens junction and the desmosome which link the actin cytoskeleton and intermediate filaments to cadherin-based adhesion sites, respectively [4]. The molecules of the cadherin superfamily of cell-cell adhesion receptors include among other members also the classical cadherins (e.g. E-cadherin and N-cadherin) and the desmosomal cadherins (e.g. desmoglein-3 and desmocollin-3). These proteins are single-span transmembrane proteins which all possess extracellular cadherin (EC) repeats. These EC repeats are capable of calcium binding and mediate the interaction capacity of the extracellular domain [5]–[7]. The differences in the structures of the cytoplasmic domains of the desmosomal and classical cadherins enable interactions with specific intracellular binding partners of the catenin protein family [8]–[10]. Except for the structurally unrelated α-catenin [11], [12], the characteristic feature of the catenin protein family is the so-called armadillo (ARM) repeat domain that is formed by a series of approximately 45 amino acid long segments [13], [14]. Classical cadherins usually associate with the catenin family members β-catenin and p120catenin, whereas desmosomal cadherins preferentially bind to γ-catenin and plakophilins [10], [15].

A special member of the catenin family is γ-catenin, also known as plakoglobin, which is so far the only protein shown to be present both in the adherens junction and the desmosome [16], [17]. γ-catenin is important for the structure of the adherens junction since it is capable of substituting β-catenin by bridging the cytoplasmic domain of cadherins with α-catenin and the actin cytoskeleton [18]–[20]. Within the desmosome, γ-catenin interacts with the cytoplasmic domains of the desmosomal cadherins and links this adhesion complex to the intermediate filament binding protein desmoplakin [21].

Membrane rafts are cholesterol dependent nanoscale structures of cellular membranes. One of the most striking abilities of rafts is that they can regulate the distribution of proteins within the plasma membrane and thus form platforms for cell signaling, viral assembly and membrane trafficking (for a review, see [22]). Many cell adhesion proteins have also been shown to be associated with membrane rafts. However, the degree of association of E-cadherin with rafts appears to be highly dependent on the cell type [23]–[25]. N-cadherin also partially localizes in membrane rafts, and disruption of rafts leads to weakened cell-cell adhesion and disorganization of N-cadherin dependent cell-cell contacts [26]. Taulet *et al.* have demonstrated that membrane rafts are important for the recruitment of the small GTPase RhoA to N-cadherin-catenin complexes, regulating RhoA activity during the onset of myogenesis [27]. Thus, the association of adhesion proteins with rafts may be a general characteristic of many cellular functions and may influence the signaling and trafficking processes originating from cell-cell adhesion complexes.

Flotillin-1 and flotillin-2 are two homologous, ubiquitously expressed proteins that are tightly associated with membrane rafts [28]–[31]. Flotillins have been suggested to be involved in a plethora of cellular processes such as membrane receptor signaling, phagocytosis and endocytosis, cell-matrix adhesion and regulation of actin cytoskeleton [32]–[36]. Our recent data have revealed an important role for flotillin-1 as a regulator of epidermal growth factor receptor (EGFR) activation and as a scaffold protein for mitogen activated protein (MAP) kinase signaling [32]. Knockout mouse models for both flotillins have recently been generated, but they do not show any major developmental defects [37]–[39]. However, breeding of the flotillin-2 knockout mouse with an established breast cancer mouse model showed that the formation of lung metastases was significantly reduced upon flotillin ablation [37], implicating a functional role for flotillins in migratory processes during breast cancer progression. In addition, another study revealed that the expression level of flotillin-1 significantly correlated with clinical staging and poor breast cancer patient survival [40].

Although flotillins do not traverse the membrane, they were discovered by means of antibodies directed against cell surface proteins, leading to the assumption that flotillins play a role in cell adhesion [31], [41]. Later on, it has been shown that the antibodies used for these studies do not recognize flotillins directly but some cell surface molecules that are evidently associated with flotillins [42], [43]. Furthermore, flotillins interfere with the distribution of cell adhesion molecules in the imaginal disc of drosophila [44], and overexpressed flotillins were shown to localize to cell-cell-contact sites [25], [29], [45]. Some adhesion molecules such as intercellular adhesion molecule 5/telencephalin colocalize with flotillins in microdomains [46]. In addition, flotillin-2 coprecipitates with N- and E-cadherin [47], [48], and a stable knockdown of flotillin-1 results in an impaired recruitment of p120catenin and E-cadherin in lipid rafts in HT-29 cells [49]. Although several studies point to an involvement of flotillins in cell-cell adhesion, the molecular details have not been characterized in non-cancerous mammalian epithelial cells. Thus, this study was carried out in order to analyze the function of flotillins in epithelial cell-cell adhesion in human mammary epithelial cells. We here show that flotillin-2 influences the morphology of adherens junctions and the association of adhesion proteins with detergent insoluble microdomains. We show that flotillins directly interact with γ-catenin which is present in both adherens junctions and desmosomes. Thus, our data suggest a novel molecular mechanism how flotillins influence cell-cell adhesion of epithelial cells.

## Materials and Methods

### Generation of Plasmids

γ-catenin plasmid DNA was obtained from Addgene (plasmid 32228) and cloned into vectors pGEX4T1 (GE Healthcare) and pMALc2x (New England Biolabs) using the primers listed in Table 1. The ARM-domains within the amino acid sequence of human plakoglobin/γ-catenin (Swiss-Prot: NP_002221.1) were predicted using the SMART software (http://smart.embl-heidelberg.de/). Sequences encoding the N-terminus (NT) the NT+ ARM 1-8, ARM 1-12, ARM1-6, ARM6-12, ARM6-8, ARM8-12, ARM1-12+ C-terminus (CT) and CT were generated using the indicated primers and cloned into vector pGEX4T1 (GE Healthcare).The coding region of rat flotillin-1 (GenBank: U60976) and flotillin-2 (GenBank: AF023302) was cloned into pET41a vector (Novagen). Full length α-catenin (GenBank: NM_001903.2) cDNA was amplified from MCF-7 cDNA by standard PCR using the primers CTATAGAATTCATGAC-TGCTCATGCAGG (for) and CTATAGTCGACTTAGATGCTGTCCATAGCTTTG (rev) and cloned into vector pGEX4T1 (GE Healthcare) at EcoRI and SalI restriction sites. The full length constructs for β-catenin-pGEX5x1 (GenBank: NM_001098210.1) was a kind gift from Anna Starzinski-Powitz (University of Frankfurt, Germany). A GST-fusion construct of p120-catenin (GenBank: AF062343.1) was created by standard PCR using the primers CTATAGAATTCATGGACGACTCAGAGG (for) and CTATAGCGGCCGCCTAAATCTTCT-GCATCAAGGGTGTTG (rev) followed by cloning into vector pGEX4T1 at EcoRI and NotI restriction sites.

**Table 1:** Primers used for the generation of the γ-catenin constructs.

Construct	Primer (5′ to 3′)
γ-cat FL GST	CTATAGGATCCATGGAGGTGATGACCCTGATG (for)
	CTATAGAATTCCTAGGCCAGCATGTGGTCTGC (rev)
γ-cat FL MBP	CTATAGGATCCATGGAGGTGATGACCCTGATG (for)
	CTATAGTCGACCTAGGCCAGCATGTGGTCTGC (rev)
γ-cat NT GST	CTATAGGATCCATGGAGGTGATGACCCTGATG (for)
	CTATAGAATTCCTAGATGAGATGCACAATGGCCG (rev)
γ-cat NT+ARM1-8 GST	CTATAGGATCCATGGAGGTGATGACCCTGATG (for)
	CTATAGAATTCCTACTCAGGGTGGCGGCTAGTG (rev)
γ-cat ARM1-12 GST	CTATAGGATCCATGAACTACCAGGACGATGCCG (for)
	CTATAGAATTCCTACTCGGAGATGCGGAACAGG (rev)
γ-cat ARM1-6 GST	CTATAGGATCCATGAACTACCAGGACGATGCCG (for)
	CTATAGAATTCCTAATCTGAGAGGTTGCGCAGGG (rev)
γ-cat ARM6-12 GST	CTATAGGATCCTGTCCCAGCAATAAGCCTGCC (for)
	CTATAGAATTCCTACTCGGAGATGCGGAACAGG (rev)
γ-cat ARM6-8 GST	CTATAGGATCCTGTCCCAGCAATAAGCCTGCC (for)
	CTATAGAATTCCTACTCAGGGTGGCGGCTAGTG (rev)
γ-cat ARM8-12 GST	CTATAGGATCCAACAGCAAGAACAAGACGCTGG (for)
	CTATAGAATTCCTACTCGGAGATGCGGAACAGG (rev)
γ-cat ARM1-12+CT GST	CTATAGGATCCATGAACTACCAGGACGATGCCG (for)
	CTATAGAATCCCTAGGCCAGCATGTGGTCTGC (rev)
γ-cat CT GST	CTATAGGATCCATGGACAAGAACCCAGACTACCG (for)
	CTATAGAATCCCTAGGCCAGCATGTGGTCTGC (rev)

Abbreviations: γ-cat = γ-catenin; FL = full length; NT = aminoterminus; ARM = armadillo domain; CT = carboxyterminus; GST = glutathione S-transferase; MBP = maltose binding protein.

### Cell Culture and RNA Interference

MCF10A [50] and MCF7 cells [51] were obtained from Nancy Hynes (Basel, Ch), HaCaT cells [52] from P. Boukamp (Heidelberg, De) and all other cell lines from American Type Culture Collection. MCF10A cells were cultured in Dulbecco’s Modified Eagle’s Medium Nutrient Mixture F-12 (DMEM/F-12, Invitrogen) supplemented with 5% horse serum, 1% penicillin/streptomycin, 10 µg/ml insulin (Sanofi Aventis), 20 ng/ml human recombinant EGF (Sigma-Aldrich), 1 µM dexamethasone (Sigma-Aldrich), 100 ng/ml cholera toxin (from *vibrio cholerae*, Sigma-Aldrich) at 37°C under 5% CO_2_. MCF-7, HeLa, A431 and Hep3B cells were cultured in DMEM (Invitrogen/Gibco) supplemented with 10% fetal calf serum and 1% penicillin/streptomycin at 37°C under 8% CO_2_. For HaCaT cells, DMEM was supplemented with 1% sodium pyruvate and 1% non-essential amino acids. Expression of flotillin-1 and flotillin-2 was stably knocked down in MCF10A cells using the Mission Lentiviral *sh*RNA system (Sigma-Aldrich), with two viruses each targeting different sequences in human flotillin-1 or flotillin-2. The control cells were established using a lentivirus that does not target any human gene. Establishment of stable knockdown cell lines was done as described previously for HeLa cells [53].

### Antibodies

Rabbit polyclonal antibody against flotillin-1 and flotillin-2 used for immunoprecipitation and immunofluorescence were purchased from Sigma-Aldrich. Mouse monoclonal antibodies against flotillin-1, flotillin-2, E-cadherin, N-cadherin, β-catenin, γ-catenin and desmoglein-1 were purchased from BD Transduction Laboratories. The mouse monoclonal desmoglein-3 antibody was purchased from AbD Serotec. A mouse monoclonal antibody against GAPDH was obtained from Abcam. For IP of γ-catenin, a rabbit polyclonal antibody was used (Cell Signaling Technology). The mouse monoclonal desmoglein-3 antibody was purchased from AbD Serotec. The primary antibodies used for immunofluorescence were detected with a Cy3-conjugated goat anti-mouse antibody (Jackson ImmunoResearch) and with an Alexa Fluor 488 donkey anti-rabbit antibody (Molecular Probes). The primary antibodies used for Western blotting were detected with an HRP-conjugated goat anti-mouse or goat anti-rabbit antibody (Dako).

### Immunofluorescence of Cells

Cells cultured on coverslips were fixed with MeOH at −20°C. Thereafter, the cells were incubated with the primary antibody in 1% BSA, washed, incubated with the Cy3 and/or Alexa Fluor 488-conjugated secondary antibody and mounted in Gel Mount (Biomeda). The samples were analyzed with a Zeiss LSM710 Confocal Laser Scanning Microscope (Carl Zeiss).

### Cell Lysis, Gel Electrophoresis and Western Blot

Cell pellets were lysed in lysis buffer (50 mM Tris-HCl pH 7.4, 150 mM NaCl, 2 mM EDTA, 1% Nonidet P-40) supplemented with protease inhibitor cocktail (Sigma-Aldrich) and lysates were cleared by centrifugation. Protein concentration was measured with the BioRad protein assay reagent. Equal protein amounts of the lysates were analyzed by SDS PAGE and Western blot.

### Immunoprecipitation

The cells were lysed on ice for 30 minutes in immunoprecipitation lysis buffer (10 mM Tris-HCl pH 8.0, 150 mM NaCl, 5 mM EDTA, 0.5% Triton X-100 and 60 mM N-octylglucoside) supplemented with protease inhibitors and cleared by centrifugation. Unspecific binding material was removed by incubating lysates with Pansorbin beads (Calbiochem). The polyclonal flotillin-1 and flotillin-2 antibody (Sigma-Aldrich) were coupled with Dynabeads protein A (Dynal), the monoclonal γ-catenin antibody was precoupled with Dynabeads protein G and combined with an amount of lysate corresponding to 750 µg of total protein. For control, a polyclonal c-myc antibody (Santa Cruz) or a monoclonal c-myc antibody (Roche) were used.

### GST Protein Expression

The fusion proteins were expressed in the *E. coli* strain Rosetta DE3. In the case of the control GST protein, the bacteria were grown at 37°C until OD_600_ 0.4–0.6 and then induced with 1 mM IPTG for 6 h at 37°C. For the γ-catenin fusion proteins, the bacteria were induced with 0.3 mM IPTG and for the flotillin fusions with 0.1 mM IPTG for 22 h at 19°C. The cells were pelleted and lysed in lysis buffer (50 mM Hepes pH 7.4, 150 mM NaCl, 1 mM EDTA, 5% glycerol, 0.1% Nonidet P-40) supplemented with 100 µg/ml lysozyme, 1.5 µM aprotinin, 23 µM leupeptin, 1.5 µM Pepstatin A, 1 mM PMSF (all from Roth) and 1 mM dithiothreitol (DTT). GST proteins from the lysates were allowed to bind to glutathione-sepharose beads (GE Healthcare), washed with phosphate buffered saline (PBS) pH 7.4 and left on the beads for GST pulldown experiments.

### Maltose Binding Protein Expression

The *E. coli* strain Rosetta was transformed with either pMalc2x or γ-catenin-pMalc2x. The bacteria were induced with 0.5 mM IPTG for 22 h at 19°C. The cells were pelleted and lysed in lysis buffer (50 mM Hepes pH 7.4, 150 mM NaCl, 1 mM EDTA, 5% glycerol, 0.1% Nonidet P-40) supplemented with 100 µg/ml lysozyme, 1.5 µM aprotinin, 23 µM leupeptin, 1.5 µM Pepstatin A, 1 mM PMSF and 1 mM DTT. MBP proteins from the lysates were allowed to bind to amylose resin beads (New England Biolabs), and washed with phosphate buffered saline (PBS) pH 7.4. Proteins were eluted with elution buffer (20 mM Tris-HCl pH 7.4, 200 mM NaCl, 1 mM EDTA, 1 mM DTT) supplemented with 10 mM D(+) maltose for 2 h at 4°C on a rotating wheel.

### GST Pulldown

MCF10A cells were lysed for 30 min on ice in lysis buffer (10 mM Tris-HCl pH 8.0, 150 mM NaCl, 5 mM EDTA, 0.5% Triton X-100 and 60 mM N-octylglucoside) supplemented with protease inhibitor cocktail and cleared by centrifugation. Cell lysates were incubated with either 5 µg of GST or GST-tagged proteins immobilized on glutathione sepharose beads over night at 4°C. The beads were washed four times with 1 ml lysis buffer, resuspended in loading buffer, boiled for 5 min at 94°C and separated by SDS PAGE.

### GST Pulldown Using Purified Proteins

Direct GST pulldown experiments were performed for 3 h on ice using 5 µg of the purified proteins (GST, flotillin-1-GST, flotillin-2-GST bound to glutathione-sepharose beads and the eluted proteins MBP and γ-catenin-MBP) in direct GST pulldown buffer (50 mM Tris-HCl pH 7.4, 150 mM NaCl, 1 mM EDTA, 1 mM DTT, 0,01% Triton X-100). Beads were washed three times with the same buffer, resuspended in loading buffer, boiled for 5 min at 94°C and separated by SDS PAGE.

### Isolation of Membrane Rafts

Membrane rafts were isolated from stable MCF10A flotillin knockdown cells cultured for 10 days according to Harder *et al.* [54]. For each raft isolation, 2.5 mg of total protein was used.

### Quantification of the Distribution of Cell-cell Adhesion Proteins

Quantification of cell border distribution of adhesion proteins was performed with Image J software using binary pictures by defining a region of interest (ROI) spanning a cell-cell border and measuring the area covered by an adhesion protein in pixel normalized to the total area (ROI). Data are shown as the mean ± SD. Statistical comparisons were made using one-way analysis of variance (ANOVA, see below).

### Statistical Analysis

Unless otherwise stated, all experiments were performed at least three times. For the statistical analysis, Western blot bands of proteins were quantified by scanning densitometry using Quantity One software (Bio-Rad) and were normalized to GAPDH. For analyzing the lipid raft isolations, the densitometric quantification was normalized to fraction number 12. Data are shown as the mean ± SD. Statistical comparisons were made using one-way analysis of variance (ANOVA) or two-way ANOVA with Bonferroni’s multiple comparison test as appropriate using GraphPad Prism 5 software. Values of *p*<0.05 were considered significant (*), whereas values of *p*<0.01 and *p*<0.001 were defined very significant (**) and highly significant (***), respectively.

### Electronic Manipulation of Images

The images shown have in some cases as a whole been subjected to contrast or brightness adjustment. No other manipulations have been performed unless otherwise stated.

### Ethical Statement

This study does not contain any procedures requiring an ethical statement/permission.

## Results

Several studies have recently connected flotillins with breast cancer [37], [40], [55], and recent findings have strongly suggested a functional connection with cell-cell adhesion proteins and formation of adherens junctions [48]. We have here characterized the molecular function of flotillin-1 and flotillin-2 in epithelial cell-cell adhesion using the human, non-tumorigenic epithelial MCF10A cell line. Although MCF10A cells were originally derived from the mammary gland of a patient with fibrocystic disease [50], they display a polarizable epithelial phenotype [50], [56], and these cells provide a suitable, non-malignant cell culture model to study epithelial cell-cell adhesion processes.

**Figure 1:**
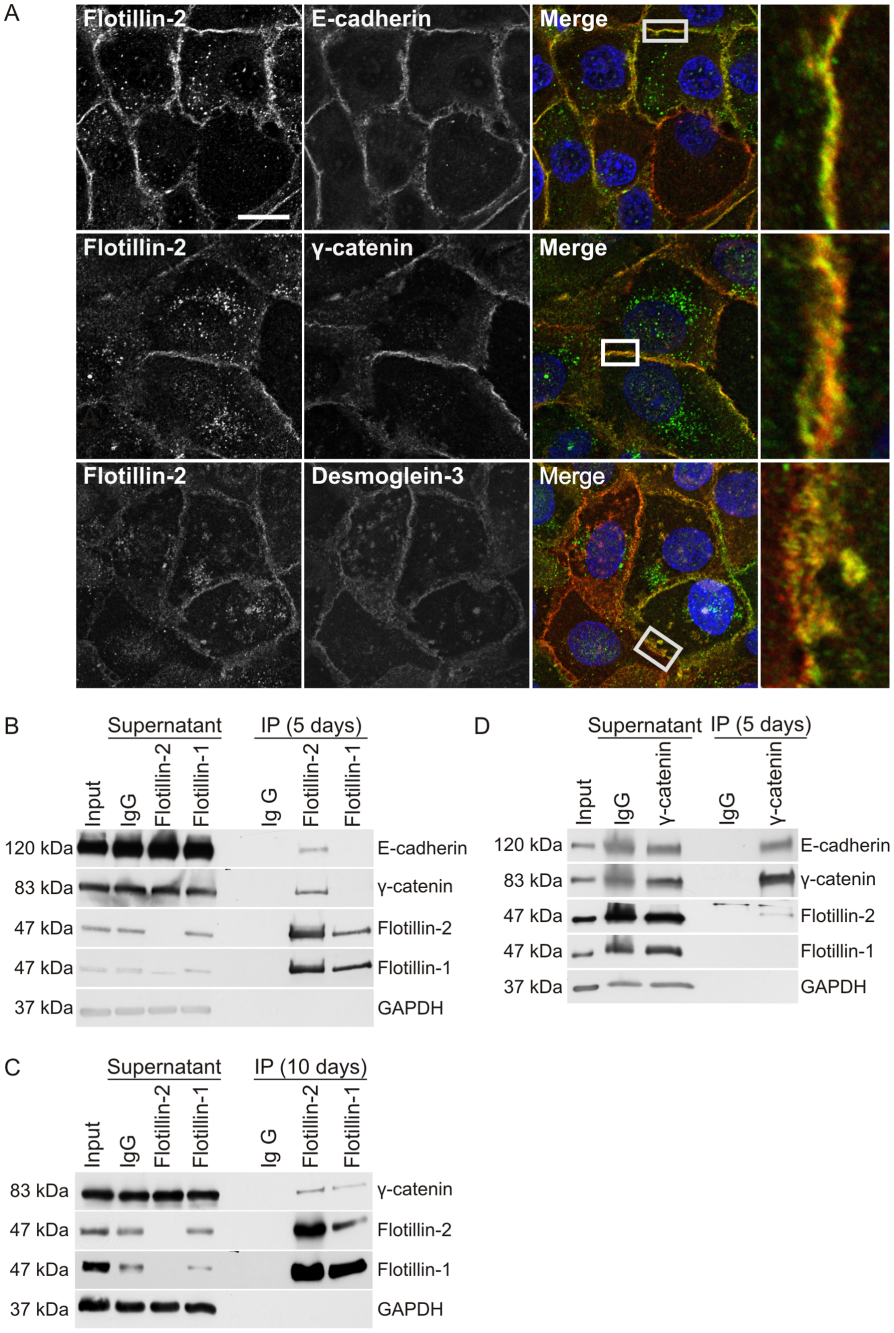
Flotillin-2 associates with core proteins of the adherens junction. (A) MCF10A cells were grown confluent for three days and stained for flotillin-2, E-cadherin γ-catenin and desmoglein-3 using specific antibodies and fluorochrome coupled secondary antibodies. Scale bar: 20 µm. (B & C) Coimmunoprecipitation of adhesion proteins with flotillins. Flotillins were precipitated with antibodies against flotillin-2 or flotillin-1 from MCF10A cells grown confluent for 5 days (B) or 10 days (C). (D) Coimmunoprecipitation of flotillins with an antibody against γ-catenin. For each experiment, 750 µg total protein was used.

Staining of subconfluent MCF10A cells (Figure S1, upper row) showed that flotillins are mainly localized in intracellular vesicular compartments, whereas in confluent cells, they preferentially reside at the plasma membrane with only little intracellular staining (Figure S1, lower row). In accordance with the data by Guillaume *et al.* in mesenchymal cells and breast cancer cells [48], flotillin-2 was found to colocalize with E-cadherin and γ-catenin at the cell-cell contact sites of confluent MCF10A cells grown on coverslips for 3 days (Figure 1A). In addition, flotillin-2 also colocalized, albeit not as strongly, with desmoglein-3, a cadherin protein of the desmosomes (Figure 1A, lowermost row). Since flotillin-2 strongly colocalized with cell-cell junctions, this suggested that flotillins might be found in a complex with cell adhesion proteins in confluent MCF10A cells. To study this, we performed coimmunoprecipitation experiments. When MCF10A cells were grown as dense monolayers for 5 days, we observed a coprecipitation of E-cadherin and γ-catenin with flotillin-2 but not with flotillin-1 (Figure 1B). However, after 10 days of culturing, during which adhesion structures fully maturate, we observed a coprecipitation of γ-catenin also with flotillin-1 in highly confluent cells (Figure 1C), implicating that it may associate with adhesion proteins at a later state of maturation as compared to flotillin-2. When γ-catenin was immunoprecipitated, E-cadherin and a fraction of flotillin-2, but not flotillin-1, could be detected (Figure 1D).

**Figure 2:**
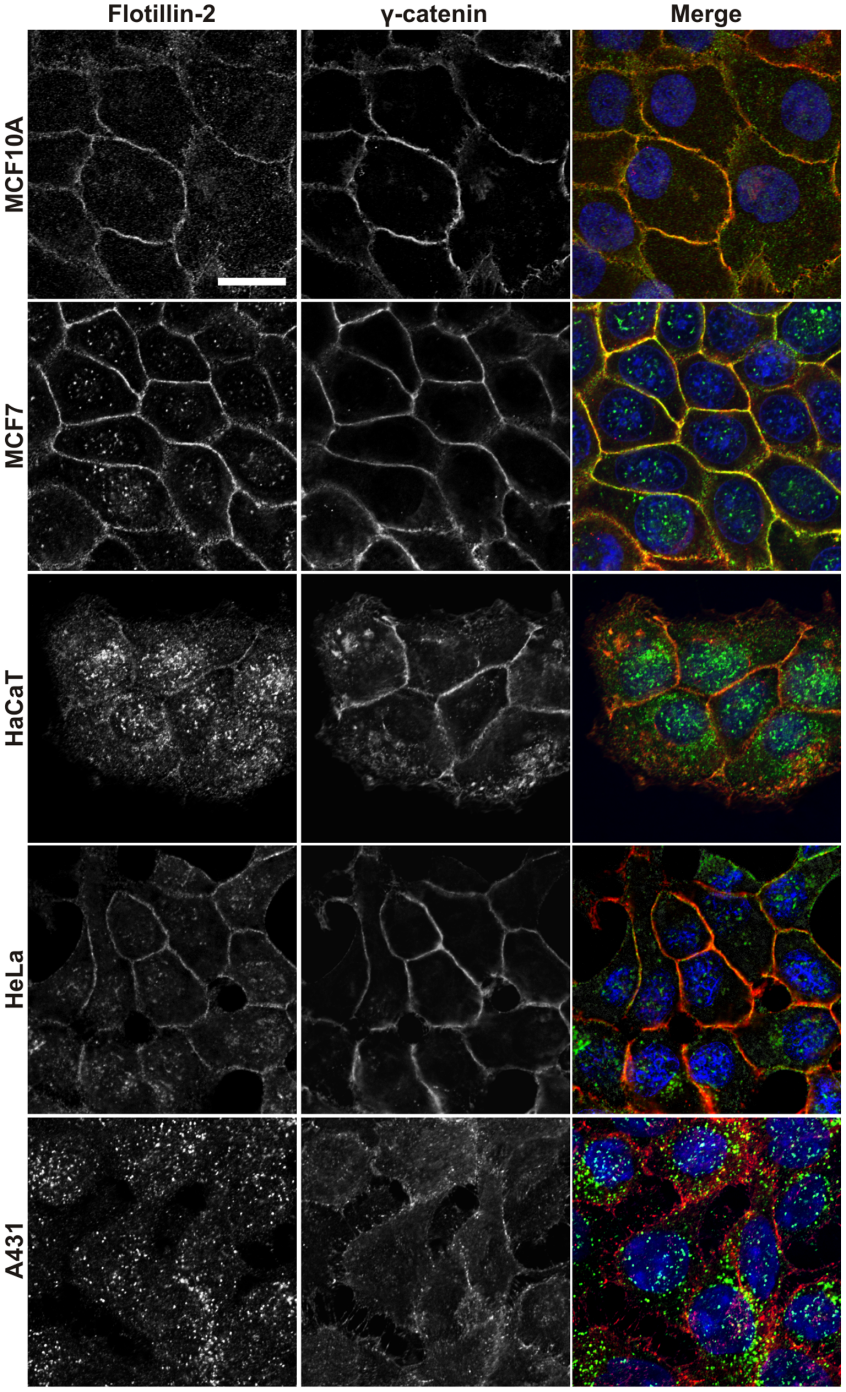
Localization of flotillin-2 and γ-catenin in various human cell lines. Staining of flotillin-2 (left column) and γ-catenin (middle column) with specific antibodies in human non-tumorigenic epithelial MCF10A cells, human breast adenocarcinoma MCF7 cells, human HaCaT keratinocytes, human cervical cancer HeLa cells and human epidermoid carcinoma A431 cells. Scale bar: 20 µm.

Since γ-catenin has previously not been connected with flotillins, we studied the colocalization of γ-catenin and flotillin-2 in various cell lines (Figure 2). Flotillin-2 was found to colocalize with γ-catenin at cell-cell borders in MCF10A, MCF7 and HeLa cells, whereas in subconfluent HaCaT keratinocytes, a lower degree of colocalization was detected due to a large fraction of flotillin-2 localized in vesicular structures in these cells. The best colocalization was observed with the cells located in the inner part of a cell patch, again suggesting that maturation of adhesion structures facilitates the colocalization of flotillins with adhesion proteins. In A431 cells, flotillin-2 was almost exclusively intracellular/vesicular, thus exhibiting no overlap with γ-catenin which showed a disorganized localization at the cell-cell borders. The localization of flotillin-2 to cell-cell junctions is calcium dependent, and calcium treatment of various cell lines resulted in uptake of flotillin-2 from the plasma membrane into intracellular vesicular structures (Data not shown). In fact, Guillaume *et al.* have shown that flotillin localization to adherens junctions may even be dependent on E-cadherin expression [48].

In accordance with the colocalization data, coprecipitation of cell adhesion proteins with flotillin-2 was detected in cells lines of epithelial origin (Figure S2). E-cadherin and γ-catenin were coprecipitated with flotillin-2, but again not with flotillin-1, from MCF7, HaCaT and Hep3B cells (Figure S2A–C), whereas in HeLa cells which express N-cadherin, γ-catenin but not N-cadherin was found in flotillin-2 immunoprecipitates (Figure S2D). The amount of coprecipitation of flotillin-2 with γ-catenin correlates well with the degree of their colocalization, as only a fraction of flotillin-2 appears to be present in the adhesion structures.

**Figure 3:**
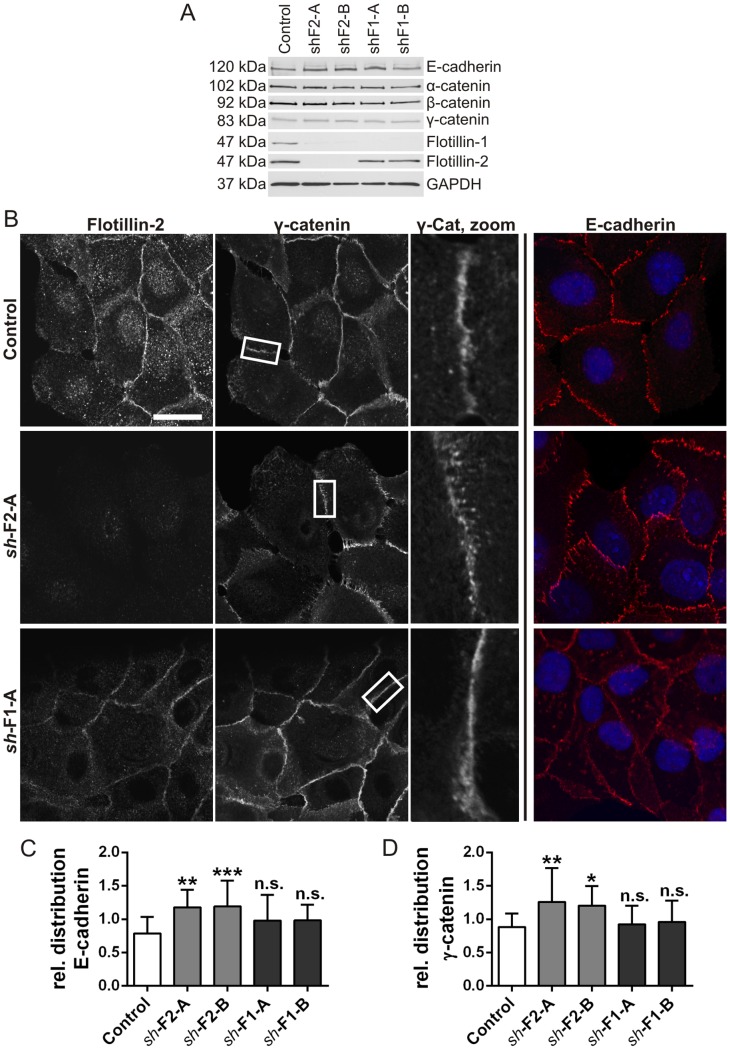
Localization of E-cadherin and γ-catenin is altered in flotillin-2 depleted MCF10A cells. (A) The expression level of various cell-cell contact proteins is not altered in MCF10A cells depleted of flotillin-1 (*sh*-F1-A/B) or flotillin-2 (*sh*-F2-A/B). Flotillins were stably knocked down in MCF10A cells by means of lentiviruses carrying respective *sh*RNAs. Expression of indicated proteins was studied in cell lysates by menas of Western blot. (B) MCF10A cells depleted of flotillin-1 (*sh*-F1-A) or flotillin-2 (*sh*-F2-A) were grown for 3 days on coverslips, fixed and stained with antibodies against flotillin-2, E-cadherin and γ-catenin. Scale bar: 20 µm. (C) and (D) Quantification of relative cell border distribution of E-cadherin and γ-catenin in MCF10A cells depleted of flotillin-1 (*sh*-F1-A/B) or flotillin-2 (*sh*-F2-A/B). Data points represent the mean ± SD of three independent experiments in which 20 cell-cell borders were measured. One-way ANOVA with Bonferroni’s multiple comparison test. *, p<0.05; **, p<0.01; ***, p<0.001.

To study the effect of flotillin depletion on cell-cell adhesion, we generated stable MCF10A cell lines in which flotillins were knocked down by means of lentiviral *sh*RNAs that we have characterized before [36], [53]. We obtained a knockdown efficiency of 80–90% for flotillin-1 and 85–95% for flotillin-2 (Figure 3A). Please note that flotillin-2 knockdown results in nonexpression of flotillin-1, whereas flotillin-1 knockdown cells show an almost normal amount of flotillin-2. Staining with flotillin antibodies demonstrated that virtually all cells exhibited the expected knockdown (Figure S3, compare with Figure 2). Microscopic analysis revealed that flotillin-1 depletion leads to a confined localization of flotillin-2 at the plasma membrane and an exclusion of flotillin-2 from intracellular vesicular compartments, whereas in flotillin-2 depleted cells, we were not able to detect any flotillin-1 staining (Figure S3).

The protein amount of E-cadherin, α-, β- and γ-catenin was not changed upon stable flotillin knockdown (Figure 3A), and densitometric quantification of Western blots revealed no significant changes in the expression of any of these proteins (Data not shown). However, the localization of γ-catenin, which is found in both desmosomes and adherens junctions, and E-cadherin, which is a *bona fide* adherens junction protein, at the cell-cell borders of MCF10A cells was altered after flotillin-2 depletion (Figure 3B, middle row). The staining for these proteins appeared to become more ragged and dispersed in the absence of flotillin-2. However, flotillin-1 depletion did not result in evident changes in the localization of these proteins (Figure 3B, lowermost row). Stainings of stable knockdown cells generated with the second *sh*RNA for each flotillin are shown in Figure S4. Quantification of the relative distribution of E-cadherin and γ-catenin at the cell-cell borders showed a significantly broader distribution upon flotillin-2 depletion (Figure 3C–D). Taken together, the alterations in the localization of adherens junction proteins upon flotillin-2 depletion point to a role for flotillin-2 in the regulation of cell-cell adhesion structures in epithelial cells, which is likely to be different from the suggested role in E-cadherin recycling in cancer cells [25]. On the basis of our data, however, it is not possible to say if the formation, stability or maintenance of adhesion junctions is affected. However, the observed changes upon flotillin-2 knockdown are well in accordance with the data of Guillaume *et al.* [48].

**Figure 4:**
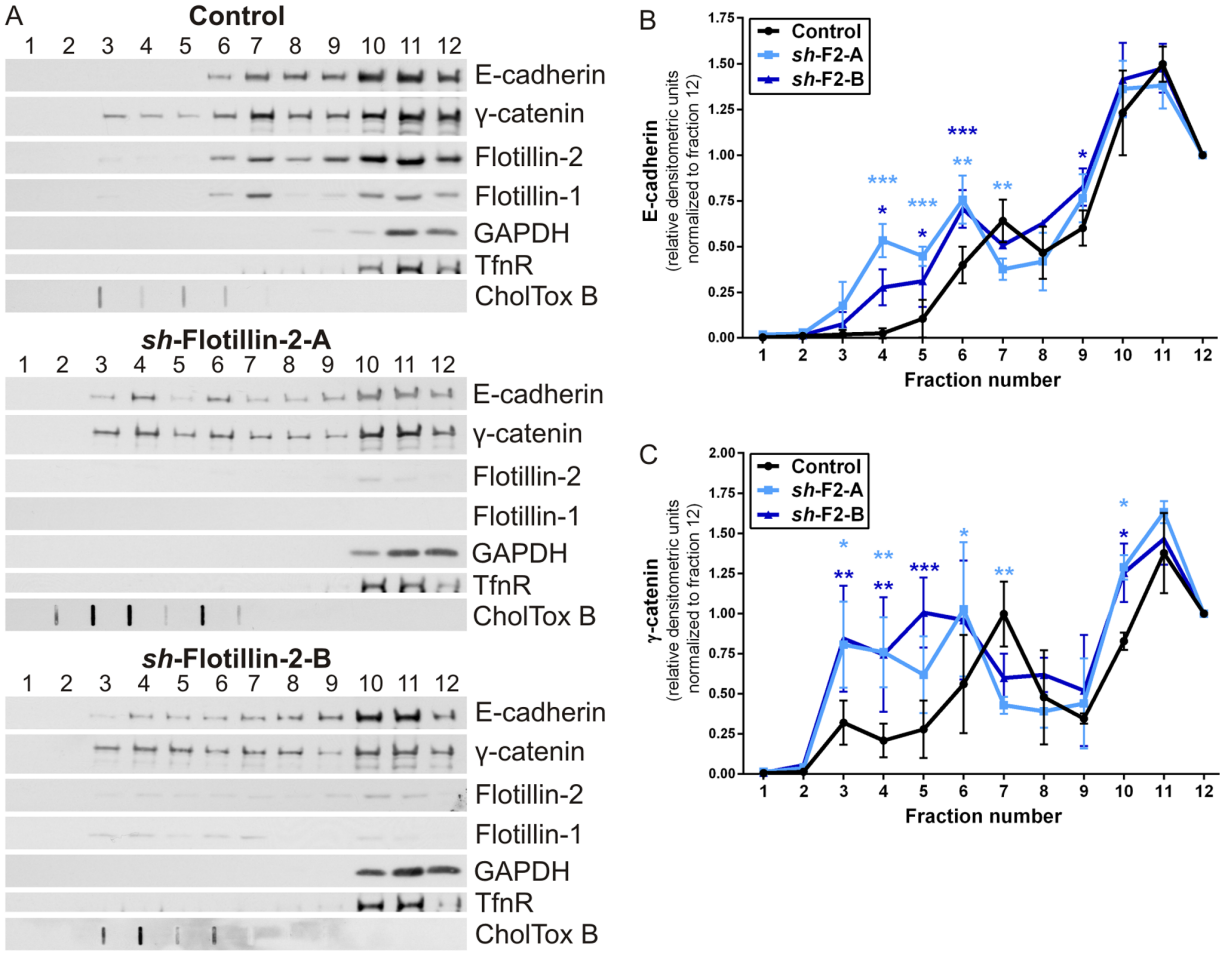
Flotillin-2 knockdown increases raft association of E-cadherin and γ-catenin in MCF10A cells. (A) Lipid rafts were isolated using detergent extraction from MCF10A control and flotillin-2 knockdown (*sh*-F2-A/B) cells after 10 days of confluent growth. Fractions 1–12 were stained with antibodies against E-cadherin, γ-catenin, flotillin-2, flotillin-1, GAPDH and transferrin receptor (TfnR). Lipid raft fractions were detected with an HRP-coupled cholera toxin subunit B. (B–C) Densitometric quantification of E-cadherin (B) and γ-catenin (C) distribution in the fractions. The signals were normalized to fraction number 12. Data points represent the mean ± SD of three independent experiments. Two-way ANOVA with Bonferroni’s multiple comparison test. *, p<0.05; **, p<0.01; ***, p<0.001.

Many cell-cell adhesion proteins have been shown to partially localize to membrane rafts in which they participate in signaling and associate with their interaction partners [26], [27], [49], [57]. Since flotillin-2 depletion affected the morphology of MCF10A cell-cell adhesions, suggesting an important functional role for flotillin-2 in epithelial morphology, we studied if the membrane raft association of E-cadherin and γ-catenin is dependent on flotillin expression in stable MCF10A flotillin knockdown cells (Figure 4). Only a fraction of flotillins was typically observed in the raft fractions, whereas the rest was found in more dense fractions. This is most likely due to the strong association of flotillins with the cytoskeleton and consistent with our earlier findings [32]. A minor fraction of E-cadherin and γ-catenin was found within the raft fractions 3–6 in control cells grown confluent for 10 days (Figure 4A, uppermost panel). Curiously, upon depletion of flotillin-2, we observed a shift of a higher amount of E-cadherin and γ-catenin into the raft fractions (Figure 4A, middle and lowermost panel), and quantification of E-cadherin (Figure 4B) and γ-catenin (Figure 4C) amounts in the fractions showed that the shift in their localization was significant. On the contrary, no change was detected in flotillin-1 knockdown cells (Figure S4A–C), in line with our results showing that flotillin-1 depletion did not significantly affect the morphology of adherens junctions. In accordance with the data of Ludwig *et al.* [38], flotillin-1 depletion led to an exclusion of flotillin-2 from the raft fractions and concentration in the non-raft fractions 10–12 (Figure S4A). These observations point to a functional role of flotillin-2 in the regulation of raft association of cell-cell adhesion proteins, which in turn might affect signaling pathways originating from cell adhesion receptors.

**Figure 5:**
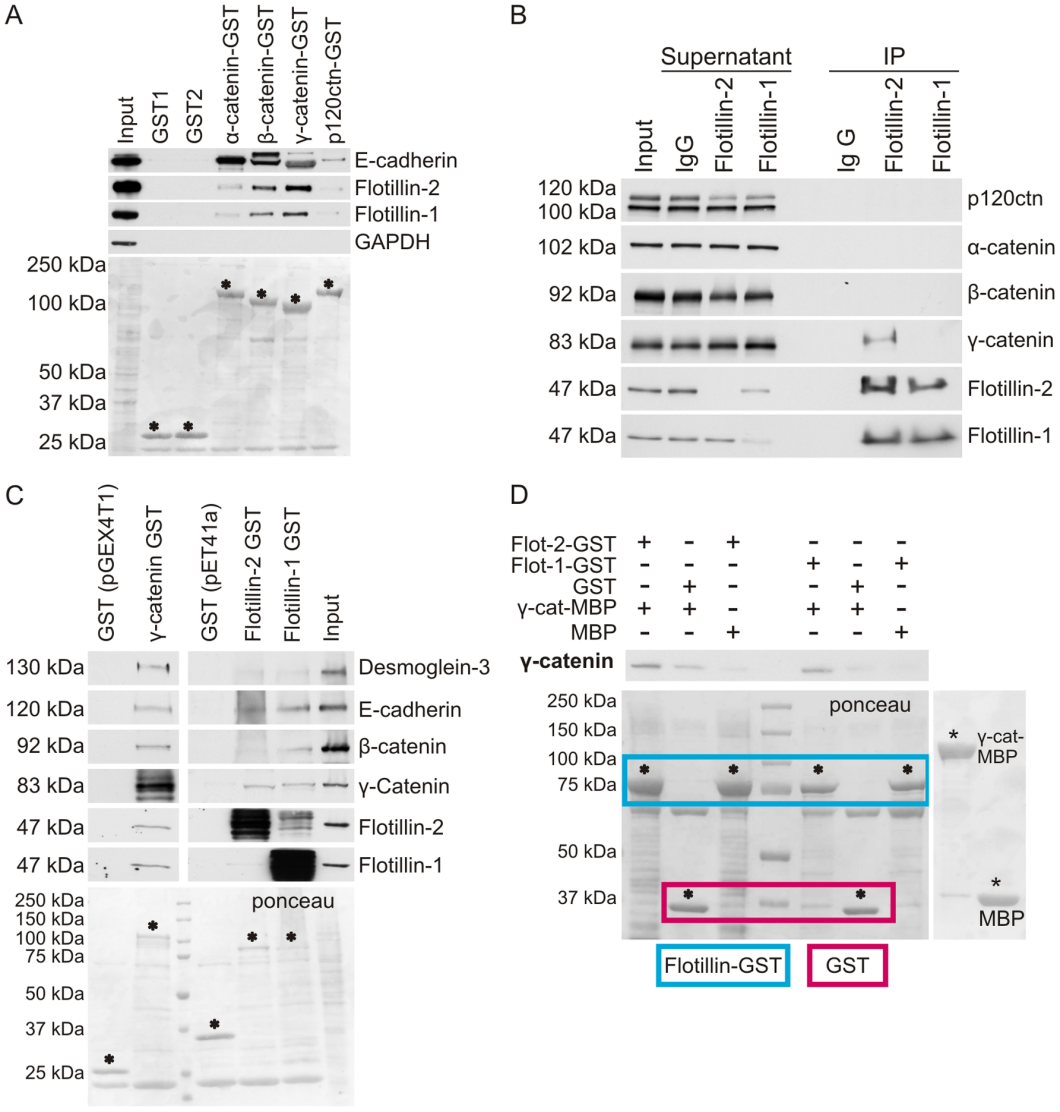
Flotillin-1 and flotillin-2 directly interact with γ-catenin *in vitro*. (A) Indirect GST pulldown from MCF10A lysate with 5 µg of purified α-catenin-GST, β-catenin-GST, γ-catenin-GST and p120-catenin-GST. Control: GST (pGEX4T1 and pGEX5x1). For each indirect pulldown, 1.2 mg of total protein was used. Asterisks mark the purified full-length proteins used in the experiment. (B) Coimmunoprecipitation with antibodies against flotillin-2 and flotillin-1 in MCF7 cells grown confluent for 5 days. For each IP, 750 µg total protein was used. (C) Indirect GST pulldown from MCF10A lysate with 5 µg purified flotillin-1-GST, flotillin-2-GST and γ-catenin-GST. Control: GST. For each indirect pulldown, 1.2 mg of total protein was used. Asterisks mark purified full-length proteins used in the experiment. (D) Direct GST pulldown with 5 µg of purified flotillin-1-GST, flotillin-2-GST (framed with blue) and γ-catenin-MBP. Control: GST (framed with red) and MBP.

The catenin protein family members are characterized by the presence of ARM domains, and our above findings suggested a previously undetected connection of flotillin-2 with γ-catenin. To test if flotillin-2 can be found in complex with the members of this protein family *in vitro*, we performed GST pulldown experiments. Full-length catenins (α-, β-, γ- and p120-catenin) were purified as GST fusion proteins and the binding of flotillins from cell lysates was tested (Figure 5A). Both β- and γ-catenin showed a strong binding to flotillin-1 and flotillin-2, whereas with α-catenin and p120catenin, only a weak interaction was detected. E-cadherin was used as a positive control and exhibited a strong binding to α-, β- and γ-catenin. These *in vitro* data were corroborated with coimmunoprecipitation experiments in which flotillins were immunoprecipitated with specific antibodies from confluent cells (Fig. 5B). While γ-catenin always coprecipitated with flotillin-2, α-catenin was never present in the immunoprecipitated fraction. We detected only a faint band for p120catenin and β-catenin in approximately half of the experiments, implicating that this interaction may be very weak and not direct. However, none of the catenins was found in the immunoprecipitates of flotillin-1. The absence of γ-catenin is likely due to the fact that the cells used for this experiment were not cultured to post-confluency. However, these results indicate that the interaction of flotillin-2 with γ-catenin is specific and may be a direct one.

To further characterize the interaction of flotillins with γ-catenin, we performed GST pulldown experiments. Bacterially expressed, purified flotillin-1-GST or flotillin-2-GST were used to probe for the interaction with adhesion proteins from MCF10A cell lysates (Figure 5C). E-cadherin and γ-catenin were detected in the pulldown fraction of both flotillins. A minor fraction of β-catenin was detected in flotillin-1, but not flotillin-2 pulldowns. When γ-catenin-GST was used for the pulldown, both flotillins as well as E-cadherin and β-catenin were detected. To test if the interaction of γ-catenin with flotillins is a direct one, we used purified flotillin-1-GST or flotillin-2-GST and γ-catenin-MBP fusion proteins. Both flotillins directly interacted with γ-catenin in this *in vitro* assay (Figure 5D).

**Figure 6:**
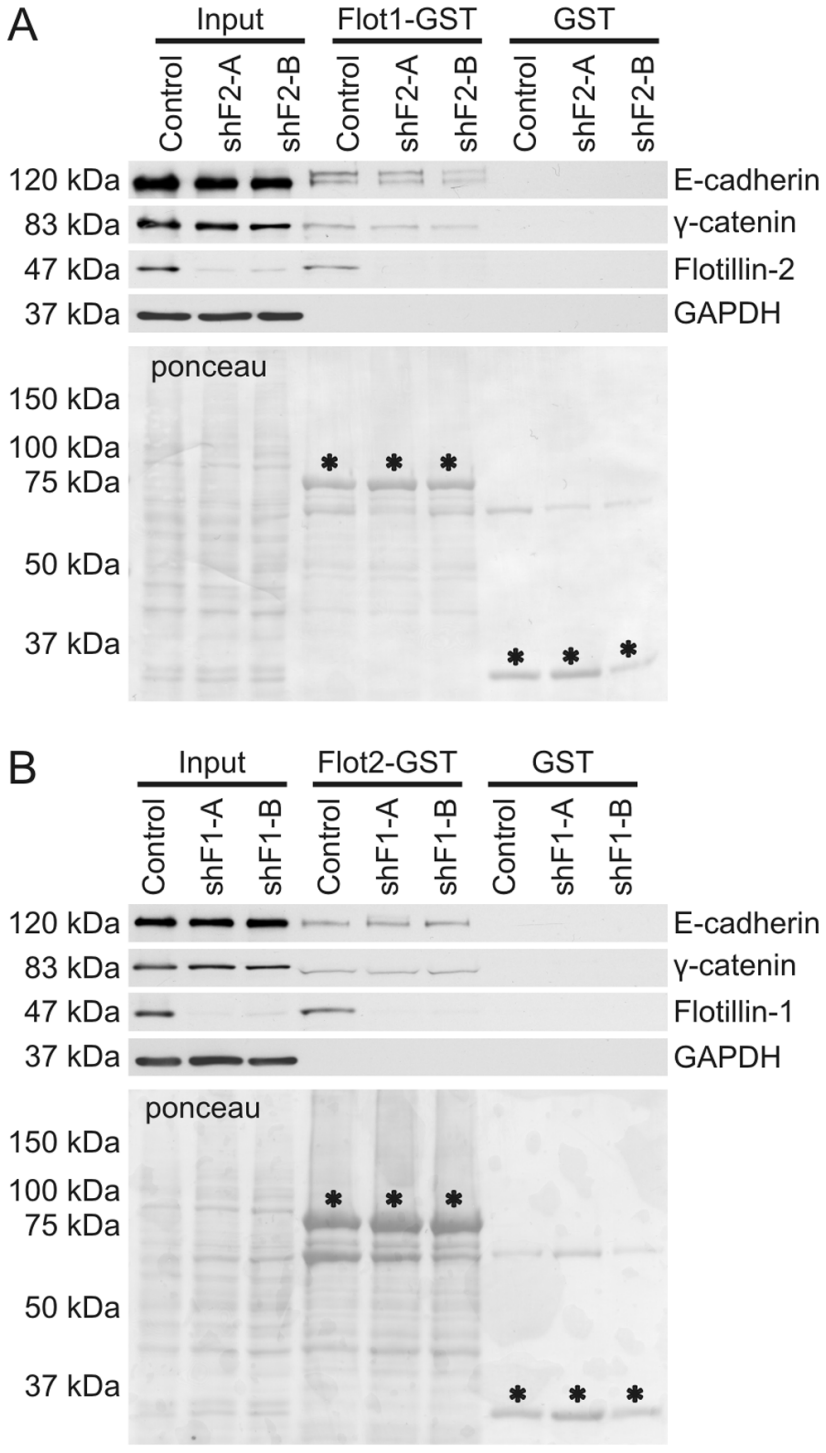
Recombinant flotillins associate with cell adhesion proteins *in vitro*. Indirect GST pulldown with flotillin-1-GST (A) or flotillin-2-GST (B) from flotillin-2 (*sh*-F2-A/B) and flotillin-1 (*sh*-F1-A/B) depleted MCF10A cells. The presence of cell adhesion proteins was detected in the pulldown fractions using specific antibodies in Western blot.

The above data suggested that flotillin-2 is more important for the cell-cell adhesion as flotillin-1 depletion did not obviously impair cell adhesion. However, although γ-catenin and E-cadherin could not be precipitated with flotillin-1 in the coimmunoprecipitation experiments, they were pulled down with flotillin-1-GST from cell lysates. Thus, the interaction with flotillin-1-GST could be indirect and mediated by flotillin-2 which is also present in the cell lysates and strongly hetero-oligomerizes with flotillin-1 [53]. Thus, we made use of the flotillin-2 knockdown cells which express very minor amounts of endogenous flotillins and tested if flotillin-1-GST would be able to pull down adhesion proteins in the absence of endogenous flotillins. Both γ-catenin and E-cadherin were detected in flotillin-1-GST pulldowns from flotillin-2 knockdown cells which are devoid of endogenous flotillins (Figure 6A). Flotillin-2-GST also interacted with both proteins from cell lysates of flotillin-1 knockdown cells which express endogenous flotillin-2 but not flotillin-1 (Figure 6B). Thus, at least *in vitro*, γ-catenin and E-cadherin are capable of interacting with both flotillins.

**Figure 7:**
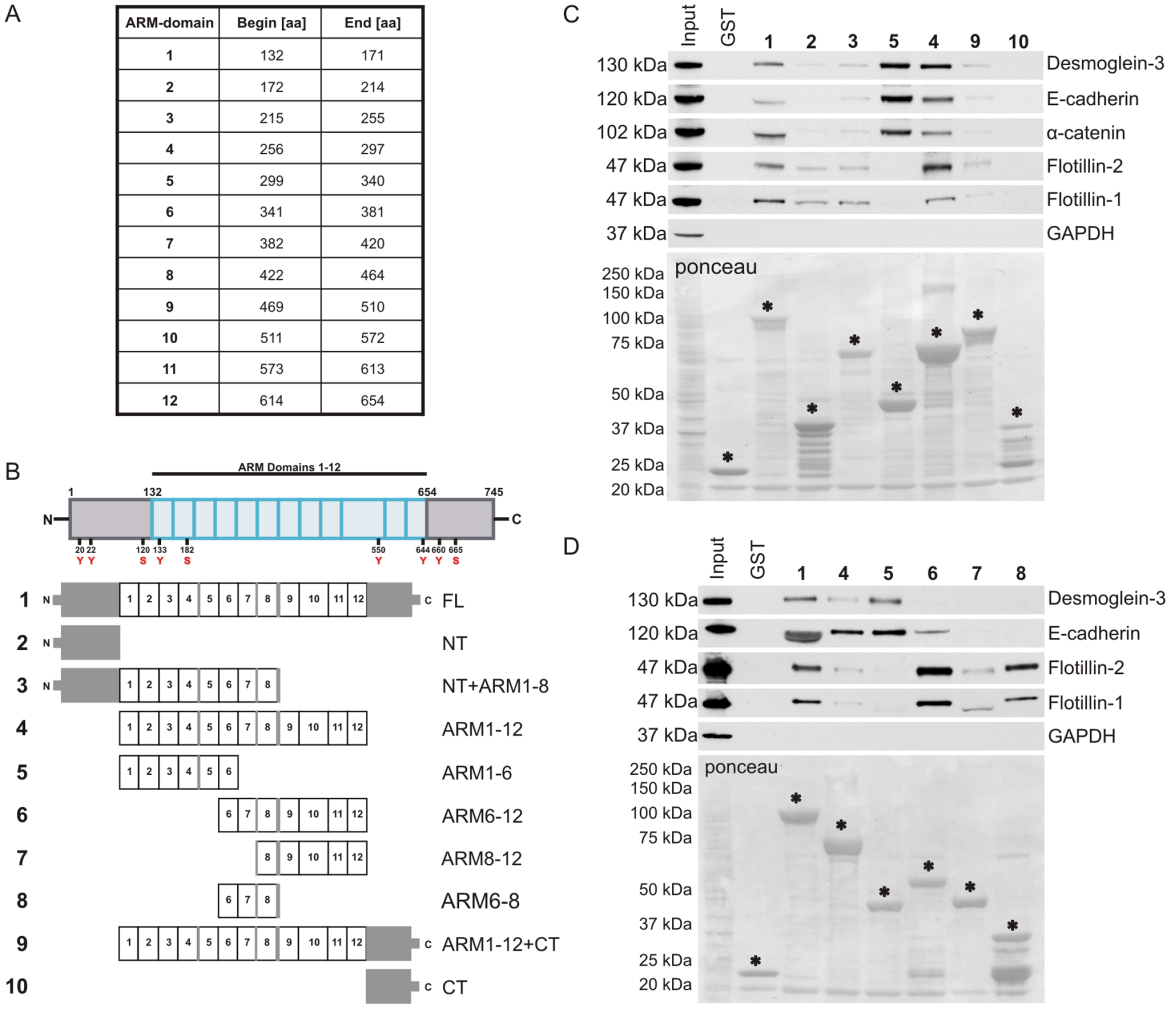
Mapping of the interaction domain of γ-catenin with flotillins. (A) Prediction of ARM domains in the amino acid sequence of human junction plakoglobin (SwissProt: NP_002221.1) with the SMART software. (B) Scheme of the generated γ-catenin constructs. NT = N-terminus, CT = C-terminus, FL = full length, ARM = Armadillo domains. (C) Indirect GST pulldown from MCF10A lysate with 5 µg of purified γ-catenin FL-GST (1), NT-GST (2), NT+ARM1-8-GST (3), ARM1-6-GST (5), ARM1-12-GST (4), ARM1-12+CT-GST (9) and CT-GST (10). (D) Indirect GST pulldown from MCF10A lysates with 5 µg of purified γ-catenin FL-GST (1), ARM1-6-GST (5), ARM6-12-GST (6), ARM8-12-GST (7), ARM1-12-GST (4), ARM6-8-GST (8). Control: GST (pGEX4T1). For each indirect pulldown, 1.25 mg of total protein was used. Asterisks mark purified full-length proteins used in the experiment.

The data above identified γ-catenin as a novel direct interaction partner of flotillins. We thus aimed at identifying the flotillin interaction domains in γ-catenin which contains 12 ARM domains (Figure 7A) flanked by amino- and carboxy-terminal (NT and CT) regions. We produced GST fusion proteins carrying different parts of γ-catenin (Figure 7B) and performed GST pulldown experiments (Figure 7C–D). E-cadherin, desmoglein-3 and α-catenin were detected in pulldowns with all γ-catenin fusion proteins containing the ARM domains 1–6, which is in accordance with the previously published interaction data on these proteins [58], [59]. However, flotillin-1 and flotillin-2 were pulled down with fusion proteins containing the ARM domains 1–8 and 1–12, but not with the one containing ARM domains 1–6 (Figure 7C). The presence of the C-terminal domain of γ-catenin diminished the interaction of the ARM domains with all four proteins tested (Figure 7C, compare lanes for constructs 4 and 9), indicating a regulatory function of the CT domain for protein binding. A weak binding of flotillin-2 to the NT domain of γ-catenin was also observed. To more precisely define the flotillin interaction domain within the γ-catenin ARM domains, additional GST fusion proteins were generated. The strongest interaction with flotillins was obtained with the proteins comprising ARM domains 6–12 or 6–8, whereas the ARM 8–12 bound to flotillins to a much lesser extent (Figure 7D). Thus, these observations suggest that flotillins bind to a discontinuous γ-catenin binding domain which consists of a major determinant around the ARM domains 6–8, most likely with a major contribution of the ARM domain 7, and possibly including the NT part of γ-catenin. The CT region may exhibit a conformational constraint that regulates the complex formation of γ-catenin with its interaction partners.

## Discussion

We here show that flotillins, especially flotillin-2, regulate cell-cell adhesion in non-cancerous mammary epithelial cells and directly interact with γ-catenin which is an important adaptor molecule that may function as a bridge between flotillins and cadherins. We have used the non-malignant, human mammary epithelial cell line MCF10A as a model system, since these cells show a good epithelial architecture and can be properly polarized as monolayers. Our data show that flotillins exert their function in cell-cell adhesion by means of e.g. affecting the association of E-cadherin and γ-catenin with detergent insoluble microdomains. Importantly, they directly interact with the catenin family members, especially γ-catenin, which in turn interacts with the core transmembrane proteins, the cadherins. Thus, these data provide a putative direct molecular mechanism that is likely to be involved in the regulation of cell adhesion by flotillins.

### Flotillins are Localized in Epithelial Adhesion Structures and Directly Interact with γ-catenin

We here show that flotillins are localized in cell-cell adhesion structures in various cell lines of epithelial origin. A colocalization was detected with the E-cadherin, a classical cadherin associated with adherens junctions, in line with the data of Guillaume et al. [48]. However, flotillins may also be involved in regulating desmosomal adhesion since they directly interact with γ-catenin which is so far the only protein that has been shown to be localized in both adherens junctions and desmosomes. In our pulldown experiments, recombinant flotillins also bound E-cadherin from cell lysates. However, this does not necessarily point to a direct interaction with flotillins, since γ-catenin was also found in the precipitated fraction and may thus function as a bridge between cadherins and flotillins. Nevertheless, the possibility that flotillins also directly interact with the proteins of the cadherin superfamily should be addressed in further studies.

Our GST pulldown results suggested that γ-catenin is able to interact with both flotillins, whereas in coimmunoprecipitation experiments, only flotillin-2 was detected in γ-catenin immunoprecipitates. In most cells, a large fraction of flotillins is associated as hetero-oligomers, but flotillin-2 also exhibits a substantial pool that is not bound to flotillin-1 [53]. Accordingly, immunoprecipitation of one flotillin usually results in coprecipitation of the other, as also observed here. Thus, the interaction of flotillins with γ-catenin in cells may rather be mediated by flotillin-2 or depend on the maturation state of the respective adhesion junction. The fact that γ-catenin also coprecipitated with flotillin-1 from cells that were grown confluent for ten days and contain more mature adhesion structures would speak for the latter possibility. However, flotillins show about 40% homology to each other, and the *in vitro* interaction of γ-catenin with flotillin-1 may also be based on the similarity to flotillin-2. We did not detect a clear effect on the morphology of cell adhesions upon flotillin-1 knockdown, implicating that flotillin-2 may indeed be more relevant for cell-cell adhesion *in vivo* (see below).

We here identified γ-catenin ARM domains 6–8 as putative flotillin binding domains. The ARM domain 7 appears to be especially important for flotillin binding, whereas the CT domain displays an inhibitory effect on flotillin/γ-catenin interaction, most likely due to conformational obstacles. Interestingly, many other adhesion proteins bind to other parts of γ-catenin and are thus not expected to compete with flotillin. Our study could verify the previously characterized binding domains of desmoglein-3 and α-catenin (ARM 1–3) as well as E-cadherin (ARM 4–5) in γ-catenin [58]–[64], which are non-overlapping with the flotillin binding region. In contrast, the major interaction site of γ-catenin with N-cadherin resides in ARM domain 7 [63], [64]. As this also appears to be the major flotillin binding site, it could explain why we were not able to coprecipitate N-cadherin with flotillins from HeLa cells. However, in mesenchymal C2C12 cells, considerable coprecipitation of flotillins with N-cadherin has recently been shown [48]. The reason for this discrepancy could be either the different cell lines used or the fact that different detergents were used for cell lysis. We have here used very stringent lysis conditions to make sure that also the detergent resistant microdomains are lysed and we only detect strong protein-protein interactions. However, this may also result in loss of weaker or indirect interactions, as may be the case between flotillins and N-cadherin.

### Functional Impairment of Cell Adhesion upon Flotillin Depletion: Putative Molecular Mechanisms

Our data show that adhesion structures containing E-cadherin and γ-catenin are morphologically altered upon flotillin-2 depletion, which results in non-expression of both flotillins, whereas barely any morphological effects were seen in the absence of flotillin-1 in MCF10A cells. On the other hand, flotillin-1 has been shown to associate with ponsin [65], which in turn directly interacts with L-afadin, a protein that is found in adhesion structures that are required for the formation of adherens junctions [66]. However, the association of flotillin-2 with the ponsin/L-afadin containing structures has not been studied, and it is not clear how interference with flotillin-2 function might affect these very early cell-cell adhesion structures.

Recently, it was suggested that flotillin-2 affects E-cadherin localization and possibly recycling by increasing the surface expression and plasma membrane associated signaling of the EGFR in A431 cells [25]. These effects were observed under EGF stimulation which has been shown to induce EMT in A431 cells [67], whereas we have here used steady-state cells which were cultured into confluency in order to allow a formation of firm cell-cell adhesion structures. In their study, Solis *et al.* stated that EGFR endocytosis is impaired upon flotillin-2 knockdown [25]. However, this appears to be a cell type specific effect, most likely due to the very high degree of overexpression of the EGFR in A431 cells. We have not observed any inhibitory effect on EGFR endocytosis upon flotillin knockdown in various cell lines ([32] and our unpublished data), including the MCF10A cells used in this study. Furthermore, in A431 cells, very little E-cadherin was observed in detergent resistant membranes, and flotillin knockdown did not influence E-cadherin association with rafts ([25] and see below). We here observed a significant increase in the raft association of both E-cadherin and γ-catenin upon flotillin-2, but not flotillin-1, knockdown. Thus, our data here provide a novel mechanism for the function of flotillin-2 in cell-cell adhesion, which is clearly different from that suggested previously by Solis *et al.* [25].

In MCF10A (this study) and MCF7 (our unpublished data) cells, E-cadherin and γ-catenin partially localize to lipid rafts. Several studies have shown a partial association of AJ proteins with rafts. In Hela cells, γ-catenin has been identified as a component of rafts [68], and the association of E-cadherin with lipid rafts was also shown in L2071 mouse fibroblasts [24]. On the other hand, although flotillin-1, flotillin-2, γ-catenin and desmoglein-2 localize to lipid raft fractions in A431 cells [23], two studies have failed to show that E-cadherin would also be present in these domains in this cell type [23], [25]. However, in human colon adenocarcinoma HT-29 cells, in which rafts are important for complex formation between p120catenin and E-cadherin, depletion of flotillin-1 leads to a diffuse localization of both proteins and impairs their recruitment in lipid rafts [49]. Thus, our observation that flotillin depletion increased the raft association of E-cadherin and γ-catenin in MCF10A cells was somewhat surprising. Recently, Guillaume *et al.* have shown that in C2C12 and MCF7 cells, flotillins and N-cadherin can be patched into microdomains containing the GM1 ganglioside by means of antibody induced crosslinking of cholera toxin B subunit. Depletion of flotillin-1 in C2C12 cells resulted in reduced copatching of overexpressed, GFP-tagged N-cadherin with cholera toxin B subunit [48]. This might imply a reduced association of N-cadherin in detergent resistant microdomains, but this was not directly assessed biochemically in the said study. We here observed an increase in the biochemical association of E-cadherin and γ-catenin with detergent insoluble domains in MCF10A cells depleted of flotillin-2. In our detergent gradients, however, the association of cholera toxin B subunit with the light fractions was not clearly altered in the absence of flotillins, implicating that in these cells, there is no functional connection between flotillins and GM1 domains. Importantly, one has to keep in mind that several raft types exist, and the effects displayed on the adhesion proteins upon flotillin depletion may be dependent on the cadherin protein analyzed and on the lipid profile of the respective cell line used.

In the absence of flotillins, the raft association of adhesion proteins was increased, which might be interpreted as flotillins displaying an inhibitory effect on the association of adhesion proteins with rafts. However, we favor another model for the function of flotillins in the regulation of adhesion. We postulate that flotillin dependent incorporation of adhesion proteins in specific “flotillin rafts” may facilitate the formation of firm adhesions by providing more time to form well organized structures in the ordered lipid environment, equal to what has been postulated for viruses that are assembled in rafts [69]. It is likely that in the absence of flotillins in MCF10A cells, adhesion proteins are recruited into other kinds of microdomains which are detergent insoluble but not capable of fully supporting the formation of adhesion structures, as suggested by our data. Intriguingly, recent findings have suggested that one function of flotillins at the plasma membrane may be to modulate the mobility of transmembrane proteins such as Alzheimer amyloid precursor protein, EGFR or the dopamine transporter [32], [70], [71], which is in line with our present data. However, our data are also fully consistent with a model that during restructuring of adhesion junctions, e.g. during EMT, flotillins may be important for adhesion based signaling, as suggested previously [25]. In polarizing and fully polarized cells, however, flotillins appear to fulfill a distinct function by influencing the formation and possibly also stability of cell adhesion structures by means of restricting the lateral motility of adhesion proteins and directly scaffolding some of the molecules, such as γ-catenin.

## Conclusions

In this study, we have provided a novel molecular mechanism for the function of flotillins in cell-cell adhesion. Although previous data have suggested that regulation of cell adhesion by flotillins would be based on their indirect effect on the trafficking of adhesion proteins, mediated by regulation of EGFR signaling, we here show that flotillins directly interact with important regulators of cell-cell adhesions, such as γ-catenin which are physically present in adhesion structures. Thus, our data point to a novel molecular mechanism of the influence of flotillins in cell-cell adhesion.

## Supporting Information

Figure S1 In subconfluent MCF10A cells, flotillin-1 and flotillin-2 are mainly localized in intracellular vesicular compartments, whereas in confluent cells, flotillin-1 and flotillin-2 mainly reside at the plasma membrane. Endogenous flotillins were immunostained with specific antibodies and fluorochrome coupled secondary antibodies. Scale bar: 20 µm.
(TIF)

Figure S2 **Interaction of flotillin-2 with γ-catenin is cell type independent.** Flotillin-1 or flotillin-2 were immunoprecipitated with specific antibodies and the coprecipitation of adhesion proteins was detected by means of Western Blot (A) MCF7, (B) HaCaT, (C) Hep3B, and (D) HeLa cells. For each immunoprecipitation, 750 µg of total protein were used.
(TIF)

Figure S3 **Localization and expression of flotillin-1 and flotillin-2 in MCF10A cells depleted of flotillins.** Stable flotillin knockdown MCF10A cells were grown on cover slips and stained with antibodies against flotillin-1 and flotillin-2. Knockdown of flotillin-1: *sh*F1-A/B, flotillin-2: *sh*F2-A/B. Scale bar: 20 µm.
(TIF)

Figure S4 **Flotillin-1 knockdown does not affect raft localization of E-cadherin and γ-catenin.** (A) Lipid rafts were isolated from MCF10A flotillin-1 knockdown cells (*sh*-F1-A/B) by means of detergent extraction after 10 days of confluent growth. Fractions 1–12 were stained with antibodies against E-cadherin, γ-catenin, flotillin-2, flotillin-1 and GAPDH. (B–C) Densitometric quantification of E-cadherin (B) and γ-catenin (C) distribution in the fractions 1–12. The signals were normalized to fraction number 12. Data points represent the mean ± SD of three independent experiments. Two-way ANOVA with Bonferroni’s multiple comparison test.
(TIF)
