# Supplementary figures and images for: Flotillins Directly Interact with γ-Catenin and Regulate Epithelial Cell-Cell Adhesion
Source: PLoS One. 2013 Dec 31;8(12):e84393. doi: 10.1371/journal.pone.0084393 (PMC3877284; doi:10.1371/journal.pone.0084393)

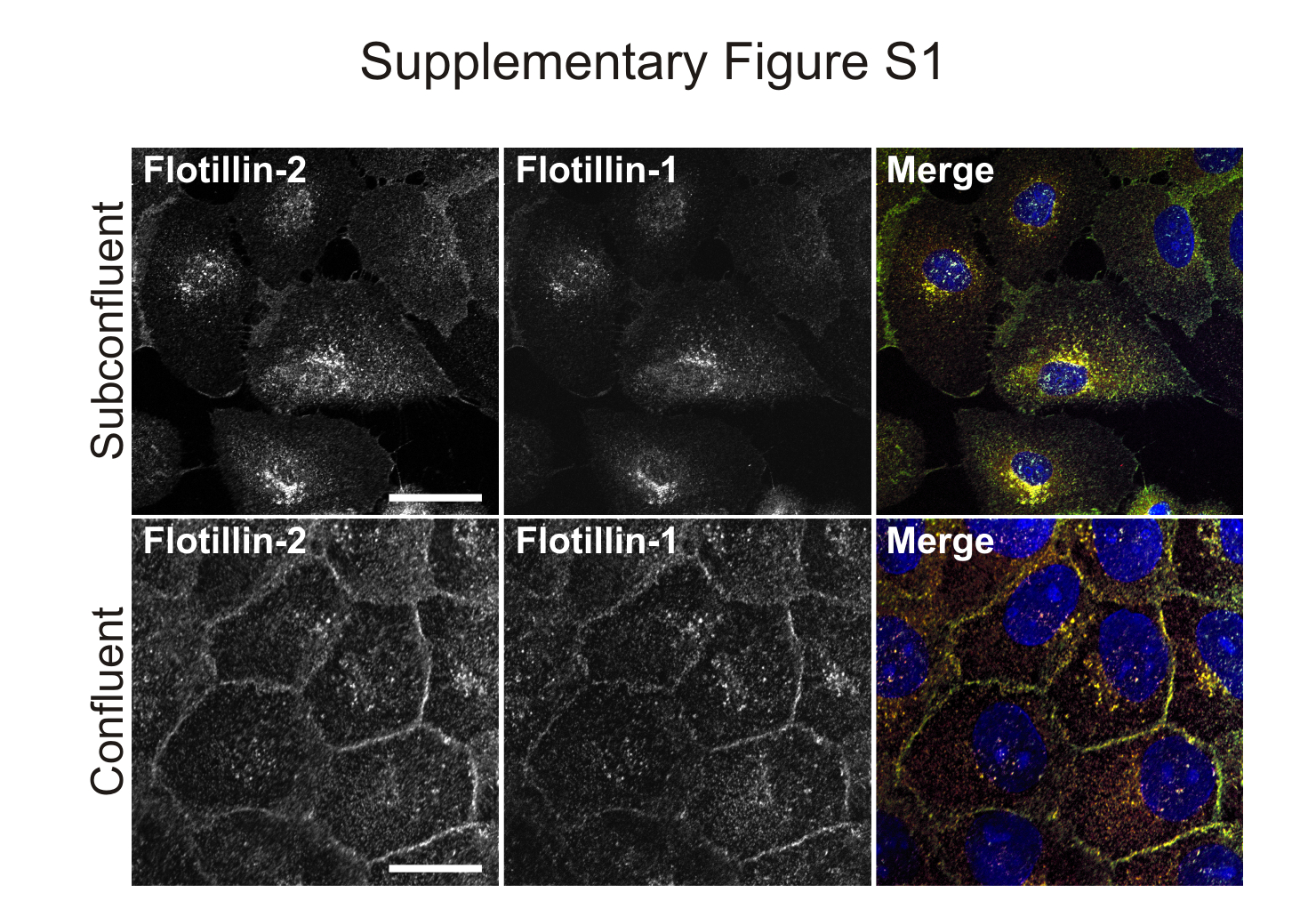

Supplement: Figure S1 — In subconfluent MCF10A cells, flotillin-1 and flotillin-2 are mainly localized in intracellular vesicular compartments, whereas in confluent cells, flotillin-1 and flotillin-2 mainly reside at the plasma membrane. Endogenous flotillins were immunostained with specific antibodies and fluorochrome coupled secondary antibodies. Scale bar: 20 µm. (TIF) [file pone.0084393.s001.tif]

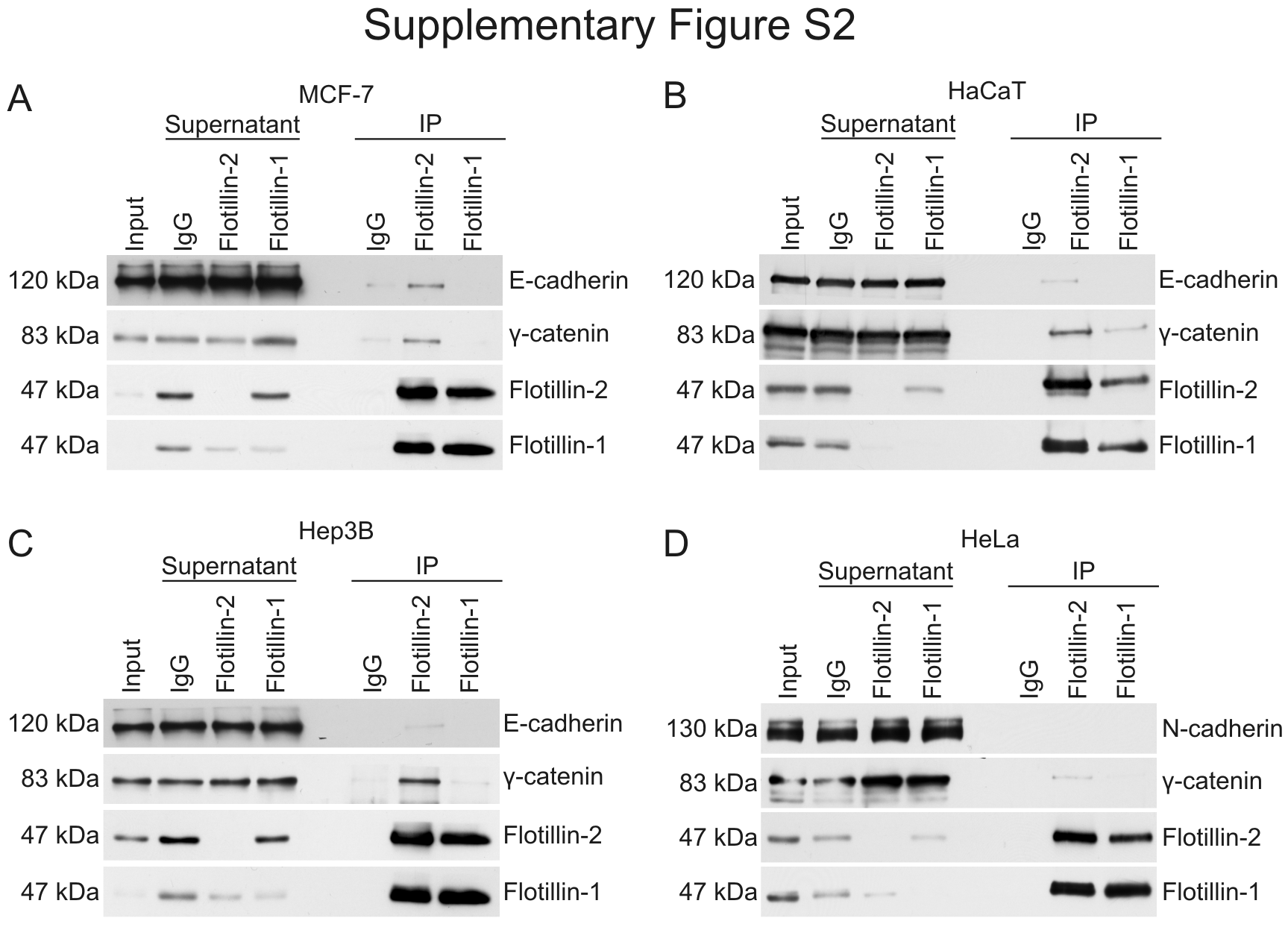

Supplement: Figure S2 — Interaction of flotillin-2 with γ-catenin is cell type independent. Flotillin-1 or flotillin-2 were immunoprecipitated with specific antibodies and the coprecipitation of adhesion proteins was detected by means of Western Blot (A) MCF7, (B) HaCaT, (C) Hep3B, and (D) HeLa cells. For each immunoprecipitation, 750 µg of total protein were used. (TIF) [file pone.0084393.s002.tif]

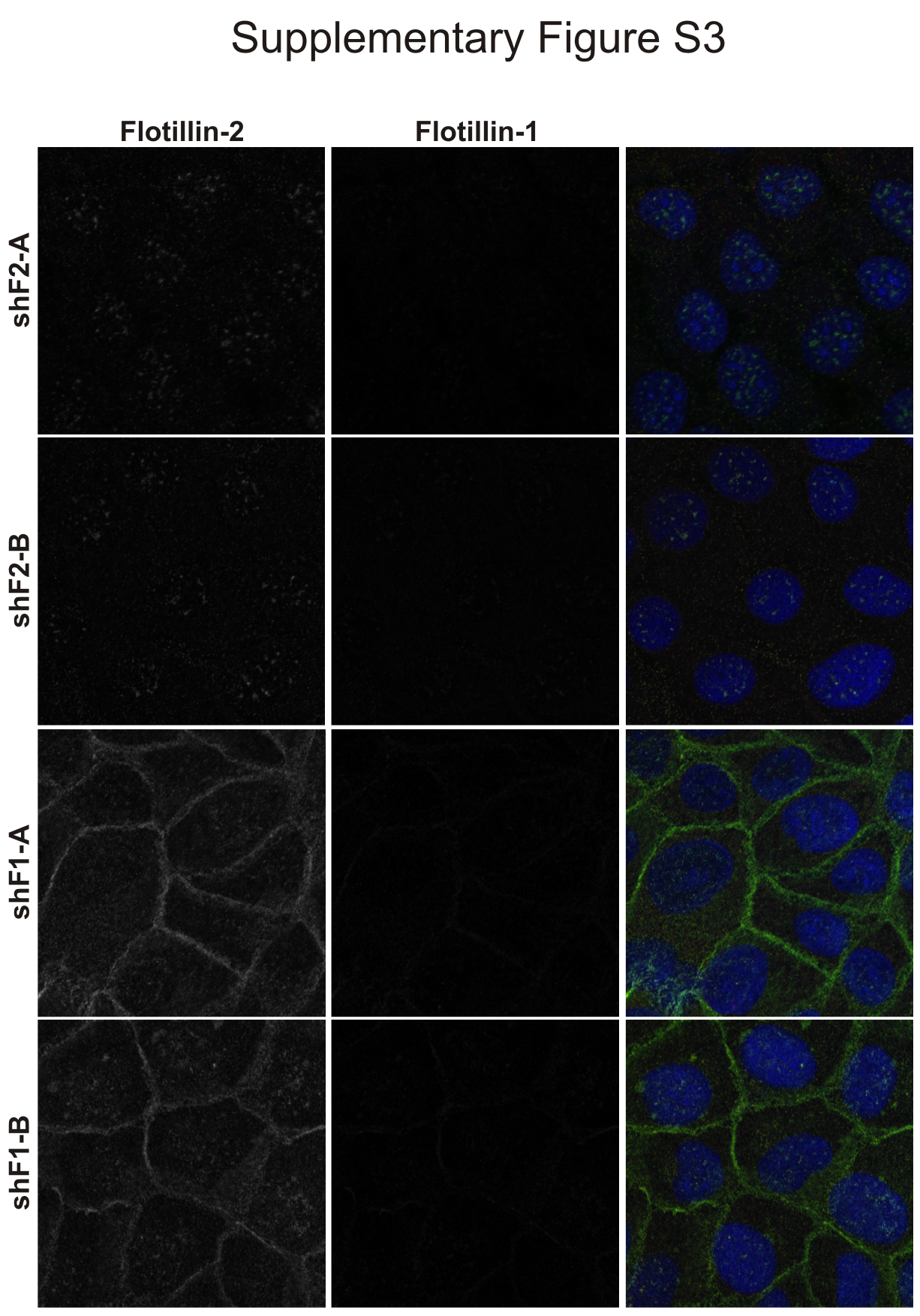

Supplement: Figure S3 — Localization and expression of flotillin-1 and flotillin-2 in MCF10A cells depleted of flotillins. Stable flotillin knockdown MCF10A cells were grown on cover slips and stained with antibodies against flotillin-1 and flotillin-2. Knockdown of flotillin-1: shF1-A/B, flotillin-2: shF2-A/B. Scale bar: 20 µm. (TIF) [file pone.0084393.s003.tif]

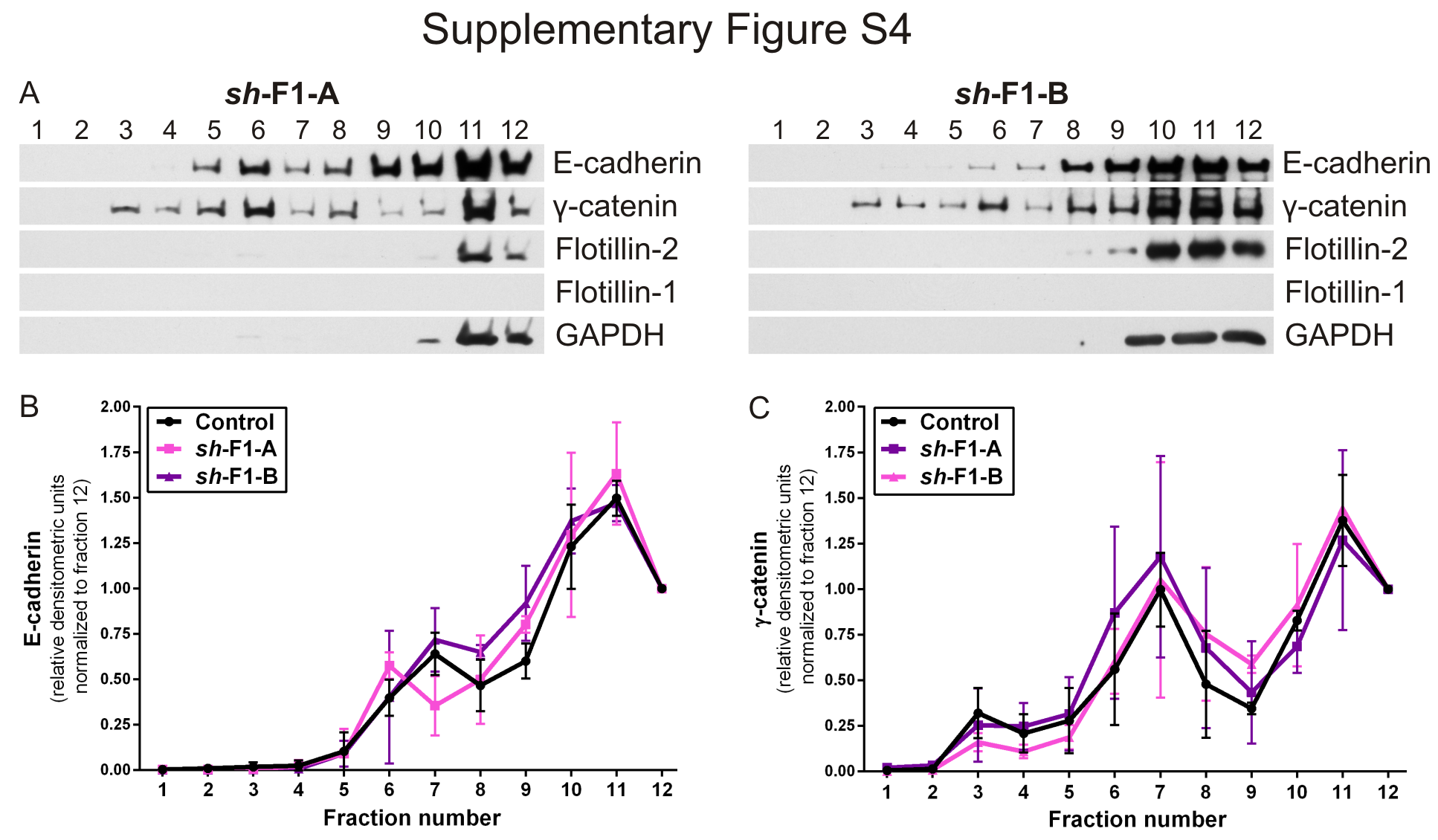

Supplement: Figure S4 — Flotillin-1 knockdown does not affect raft localization of E-cadherin and γ-catenin. (A) Lipid rafts were isolated from MCF10A flotillin-1 knockdown cells (sh-F1-A/B) by means of detergent extraction after 10 days of confluent growth. Fractions 1–12 were stained with antibodies against E-cadherin, γ-catenin, flotillin-2, flotillin-1 and GAPDH. (B–C) Densitometric quantification of E-cadherin (B) and γ-catenin (C) distribution in the fractions 1–12. The signals were normalized to fraction number 12. Data points represent the mean ± SD of three independent experiments. Two-way ANOVA with Bonferroni’s multiple comparison test. (TIF) [file pone.0084393.s004.tif]
